# The Influence of an Acute Bout of Aerobic Exercise on Vascular Endothelial Function in Moderate Stages of Chronic Kidney Disease

**DOI:** 10.3390/life12010091

**Published:** 2022-01-09

**Authors:** Jeffrey S. Forsse, Zacharias Papadakis, Matthew N. Peterson, James Kyle Taylor, Burritt W. Hess, Nicholas Schwedock, Dale C. Allison, Jackson O. Griggs, Ronald L. Wilson, Peter W. Grandjean

**Affiliations:** 1 Department of Health Human Performance and Recreation, Baylor University, Waco, TX 76706, USA; 2 Department of Sport and Exercise Sciences, Barry University, Miami Shores, FL 33161, USA; zpapadakis@barry.edu; 3 Department of Exercise Science, Lyon College, Batesville, AR 72501, USA; petersonm05@gmail.com; 4 Clinical Laboratory Science, Auburn University-Montgomery, Montgomery, AL 36117, USA; jtaylor@aum.edu; 5 Waco Family Medicine, Waco, TX 76707, USA; Burritt.Hess@wacofamilymedicine.org (B.W.H.); nschwedock@gmail.com (N.S.); jgriggs@wacofhc.org (J.O.G.); 6 Baylor Scott & White Health, Waco, TX 76712, USA; crawford.allison@me.com (D.C.A.); beardoc71@gmail.com (R.L.W.); 7 School of Applied Sciences, University of Mississippi, Oxford, MS 38677, USA; pwg@olemiss.edu

**Keywords:** high-intensity interval exercise, steady-state exercise, flow-mediated dilation, antioxidants

## Abstract

Chronic kidney disease (CKD) is directly influenced by the deleterious effects of systemic inflammation and oxidative stress. The vascular endothelium may transiently respond to aerobic exercise and improve post-exercise vascular renal function in moderate stages of CKD. Brachial artery flow-mediated dilation (FMD) is a nitric-oxide-dependent measure of endothelial function that is transiently potentiated by exercise. The purpose of the study was to determine the acute influence of a single bout of high-intensity interval exercise (HIIE) or steady-state moderate-intensity exercise (SSE) on endothelial dysfunction in moderate stages of CKD. Twenty participants (*n* = 6 men; *n* = 14 women) completed 30 min of SSE (65%) and HIIE (90:20%) of VO_2reserve_ in a randomized crossover design. FMD measurements and blood samples were obtained before, 1 h, and 24 h post-exercise. FMD responses were augmented 1 h post-exercise in both conditions (*p* < 0.005). Relative to pre-exercise measures, total antioxidant capacity increased by 4.3% 24 h post-exercise (*p* = 0.012), while paraoxonase-1 was maintained 1 h and elevated by 6.1% 24 h after SSE, but not HIIE (*p* = 0.035). In summary, FMD can be augmented by a single episode of either HIIE or SSE in moderate stages of CKD. Modest improvements were observed in antioxidant analytes, and markers of oxidative stress were blunted in response to either SSE or HIIE.

## 1. Introduction

Chronic kidney disease (CKD) affects an estimated 8 to 16% of the world’s population and 8% of the US population [1,2]. However, due to the lack of signs and symptoms in the early stages (I and II) of CKD, this number is believed to be underestimated [3]. Major risk factors for the premature development of CKD include hypertension (HTN), obesity, dyslipidemia, diabetes, sedentary lifestyle, smoking, and family history [4,5,6]. Many of these CKD risk factors create a chronic state of oxidative and nitrative stress, which can have a deleterious effect on renal and vascular function [5,7,8].

Individuals with cardiometabolic disease have decreased vascular function when compared to healthy individuals [9,10,11]. In healthy individuals, the vascular endothelium secretes many different molecules, such as nitric oxide (NO), endothelin, growth factors, adhesion molecules, and regulatory proteins with endocrine, autocrine, and paracrine functions [12]. The most common and diverse of these biomarkers is NO and its wide array of biological influences [7,8]. In conduit arteries, endothelium-derived NO regulates vasodilation, vascular tone, and inhibits platelet aggregation and adhesion characteristics [1,4,7,8,13,14,15]. In moderate stages (IIIa and b) of CKD, the vascular endothelium exhibits characteristics of endothelial dysfunction due to decrements in NO signaling and overall responsiveness of the endothelium [7,9,16]. The resulting decline in NO has a significant negative impact on renal health and function due to the high vascularization of the kidneys and high amounts of NO production [9,17]. Therefore, NO production in the kidneys decreases as the severity of the disease progresses [18].

Transient increases in endothelial function occur after acute bouts of aerobic exercise of varying intensities with similar durations [19,20,21,22]. Aerobic exercise modulates oxidative stress by producing antioxidants to counteract the harmful effects imparted by increased reactive oxygen species [14,23]. High-intensity interval exercise (HIIE) stimulates greater amounts of oxidative stress when compared to moderate-intensity exercise [14]. However, HIIE may also produce a higher post-exercise antioxidant response when compared to moderate-intensity exercise [23,24,25]. HIIE can potentially ameliorate oxidative and nitrative stress in the vascular endothelium in CKD, thus creating a net antioxidant environment in the hours post-exercise. These transient increases in vascular function are linked to the increased activation of NO production, stimulated by a higher amount of shear rate from increased blood flow [1,8,26,27].

Flow-mediated dilation (FMD) is a technique commonly used to assess transient changes in vascular function [28,29,30,31]. FMD is an effective method to discriminate between vascular endothelial function and dysfunction within multiple populations and study cohorts [32,33]. In healthy populations, FMD induces an average vessel diameter of approximately 8.5% [30,34,35] while in clinical populations, FMD induces varying ranges of endothelial responses, with an average vessel diameter of 4.6% [32,36,37]. Conduit artery vascular responsiveness is also diminished with chronic morbidities compared to apparently healthy individuals [38,39]. The reduced responsiveness appears to be related to the severity of the disease [40].

Currently, the influence of aerobic exercise on vascular endothelial health in moderate stages (IIIa-b) of CKD is not well studied. Van Craenenbroeck et al., 2015 [41] conducted the only study involving the use of FMD to assess changes in endothelial function with steady-state aerobic exercise (SSE) training in those diagnosed with moderate stages of CKD. Their study was designed to determine if 12 weeks of moderate-intensity home-based aerobic exercise training improved vascular endothelial function in patients with mid-spectrum CKD. FMD remained unchanged after the 12-week exercise program (4.0 ± 1.9% vs. 4.6 ± 3.0%). Currently, there is inadequate research to examine the effect of an acute bout of aerobic exercise and the transient effect on vascular endothelial function in mid-spectrum CKD.

The purpose of this investigation was to determine the transient effects of an acute bout of aerobic exercise on conduit artery vascular endothelial function in individuals with moderate-stage CKD. We hypothesized that improvements in vascular endothelial function would be evident via increases in FMD and antioxidants and decreases in markers of oxidative stress after an acute bout of aerobic exercise. A secondary hypothesis was that HIIE would have a greater influence on vascular endothelial function when compared to SSE.

## 2. Materials and Methods

### 2.1. Participant Recruitment

Participant recruitment for the study involved collaborating with multiple physicians, local medical centers, and health clinics. Individuals were contacted via email, phone calls, or physician referral. Inclusion criteria focused on moderate stages (IIIa and b) CKD and the following characteristics: (1) eGFR 59–30 mL/min/1.73 m^2^; (2) between 40 and 75 years of age; (3) overweight or moderately obese, BMI between 25 and 35 kg/m^2^, (4) engaged in approximately 90 min of leisure and/or work-related physical activity per week during the last three months; (5) non-smoker, defined as having never smoked or quit for >6 months; (6) free from uncontrolled hypertension and documented cardiovascular and pulmonary complications; (7) free from non-cardiac surgical procedures for at least two months; (8) free from musculoskeletal problems that would preclude treadmill walking/jogging, and (9) able to maintain regular and stable medication use. Individuals were excluded if (1) CKD was previously diagnosed as IgA nephropathy; (2) post-infectious glomerulonephritis; (3) HIV nephropathy; (4) focal stenosis; (5) renal artery stenosis; (6) eGFR > 60 and <30; and (7) lupus nephritis. The focus of our participant recruitment was to obtain individuals whose primary development of CKD was due to hypertension and diabetes.

**Figure 1:**
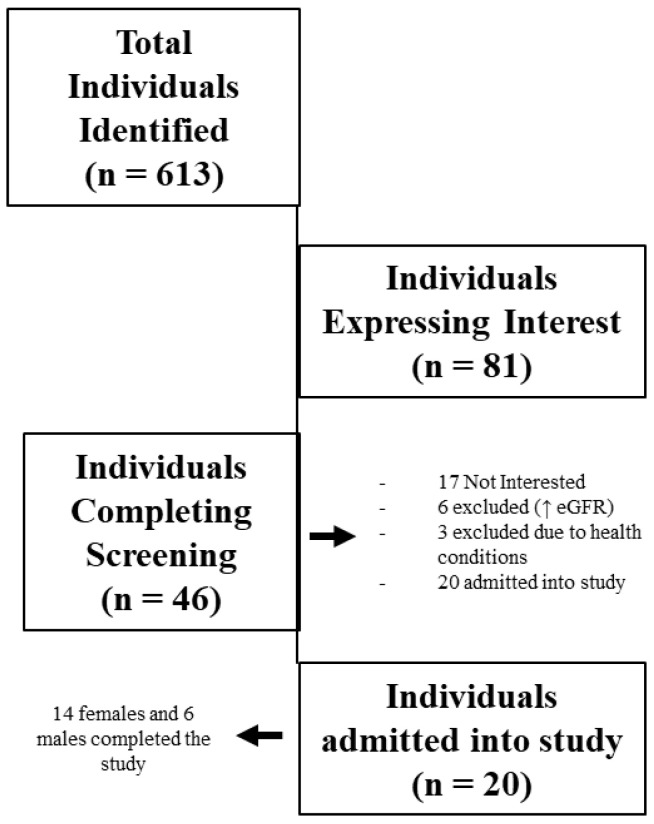
Participant recruitment, screening, and admittance into the study.

A total of 613 individuals were identified as potential research participants (see Figure 1). In total, 46 individuals met entry criteria based on the inclusion/exclusion characteristics and agreed to participate in the study. Each of the 46 participants completed the initial, preliminary, and physiologic screening sessions. However, during the physiological screening session, 26 were excluded due to higher than desired eGFR (*n* = 18), musculoskeletal concerns (*n* = 7), and positive stress tests (*n* = 1). The remaining 20 participants met all the inclusion criteria, passed the physiological screening session, were admitted into the study, and completed the study. 

### 2.2. Screening

Every participant in the study signed an informed consent document, and their physician cleared them prior to their participation. Participants were asked to seek guidance from their physician regarding the safest, lowest medication dosage that can be achieved during the experimental conditions. We were particularly interested in limiting or diminishing the effects of medication classes known to affect endothelial phenotype and vascular responses, such as lipid-lowering agents, antihypertensive, glycemic control agents, and hormone replacement therapy [42].

For screening, participants reported to the lab after an 8 to 10 h fast limited to water ingestion only. Participants brought physician release documentation and a copy of their prior blood results. Experimental procedures were reviewed again with each participant. Participants reviewed their health history, physician release and medication guidance, and prior blood record with one of our physician coinvestigators.

After the physician screening, a small blood sample was obtained and sent to a CDC-certified lab. This blood sample was used to clarify the participant’s current kidney function status via eGFR. Height, weight, and waist circumference were assessed. We also measured body composition (e.g., lean, fat, and bone tissue) by dual-energy X-ray absorptiometry (Hologic Inc., Bedford, MA, USA), followed by carotid artery thickness using ultrasound (Logic S7 Expert/Pro Ultrasound^™^, General Electric^®^ system (General Electric Company, Boston, MA, USA)).

Each participant performed a standardized maximal graded exercise test (Bruce Protocol) on a treadmill to determine hemodynamic responses to exercise of increasing intensity, as well as cardiovascular fitness. Heart rate, blood pressure, and rating of perceived exertion (RPE) were monitored throughout the test. Respiratory gasses (VO_2_ and VCO_2_) were measured continuously using a mouthpiece and headgear connected to an integrated respiratory gas analysis system (ParvoMedics, Sandy, UT, USA). The exercise test began with 2 min of warm-up at a walking pace that was comfortable for the participant. The speed and incline of the treadmill were increased every 3 min until the participant reached volitional fatigue. Exercise VO_2reserve_ (VO_2_ R = VO_2max_ − Resting VO_2_) and heart rate were used to calculate the appropriate exercise intensities for experimental exercise sessions.

### 2.3. Exercise Intervention

Experimental conditions included a single acute bout of HIIE and SSE that were matched for time and average intensity. SSE consisted of treadmill walking at a constant speed and grade to elicit 60 to 65% of VO_2_ R for 30 min (i.e., continuous, moderate-intensity exercise). In the HIIE condition, participants were asked to complete 30 min of exercise in 5 min intervals. Each interval included 3 min of fast walking/jogging on the treadmill at submaximal but vigorous exercise at 90% of VO_2_ R and 2 min of slow walking at 20% VO_2_ R (i.e., high-intensity interval exercise). Participants exercised at 60 to 65% VO_2_ R for the first and last 2 ½ minutes of this session with five, 5 min intervals in between. The high-intensity interval exercise and continuous, moderate-intensity exercise sessions were similar in duration, average intensity (60 to 65% of VO_2_ R), and caloric expenditure (HIIE 149.2 ± 43.8 and SSE 139.6 ± 40.7). Exercise conditions had a minimum of a 4-day washout period between them to prevent a potential compounding effect. Each exercise condition encompassed a total of 2 days to assess (baseline, 1-HR, and 24-HR).

### 2.4. Brachial Artery Reactivity Measurement

Ultrasound measurements of the brachial artery were obtained before exercise and again 1-HR and 24-HR after exercise for each exercise condition. To obtain these measurements, a wireless heart rate monitor (Polar H7) was used to track cardiac cycles. An automated blood pressure cuff (E 20™, Hokanson^®^, Hokanson Inc., Bellevue, WA, USA) was placed around the participant’s forearm with the proximal edge of the cuff just inferior to the medial epicondyle. Participants lay in the supine position and rested for 10 min prior to each measurement in a quiet, temperature-controlled room (20–22 °C), after which resting blood pressure was measured using a stethoscope and inflatable cuff attached to an aneroid sphygmomanometer. Next, a small amount of gel was placed on the medial side of the participant’s upper arm, and the brachial artery site was located using ultrasound (Logic S7 Expert/Pro Ultrasound^™^, General Electric^®^ system) and a 9 Hz ultrasound transducer. After obtaining a clear image of the brachial artery in B-mode grayscale, the ultrasound was switched to Doppler mode to measure blood flow velocity for 15 s. Next, the ultrasound was returned to B-mode grayscale to record baseline brachial artery diameter for 1 min. Following baseline measurements, the forearm cuff was inflated to 250 mmHg of pressure using an automated rapid inflation system. The cuff remained inflated at 250 mmHg for 5 min to occlude blood flow in the lower arm completely. After 5 min, the cuff was deflated to allow for rapid reactive blood flow through the artery. Blood flow, measured in Doppler mode, was recorded for 15 s before and after cuff deflation. Brachial artery diameter changes were recorded continuously for up to 3 min after releasing the cuff pressure. Brachial artery diameter changes and shear rates, derived from blood flow velocity recordings, were quantified offline using automated edge-detection software (Vascular Research Tools™, Medical Imaging Applications LLC^®^, Coralville, IA, USA).

### 2.5. Analysis of FMD Data

Variables from ultrasound measurements included peak changes in brachial artery diameter, indexed to initial resting vessel diameter from the pre-exercise measurement in each experimental condition, and blood flow velocity. Video recordings of vessel diameter changes were imported into analysis format using Vascular Imager software and analyzed using Brachial Artery Analyzer software (Vascular Research Tools™, Medical Imaging Applications LLC^®^, Coralville, IA, USA). Ultrasound video was slowed to six frames per second, and the diameters of the same vessel segment were quantified in millimeters and blood flow velocities in cm/s. Changes in vessel diameter were determined using flow-mediated dilation calculations according to Woodman [43] with modifications described by Pyke [44] as follows:

% Δ FMD = (BA post-occlusion − BA pre-occlusion/BA diameter pre-occlusion) × 100

where BA is the brachial artery diameter corrected for shear rate.

Pre- and post-inflation peak shear rates were estimated from blood flow using the following equation [20,25,45]:

Shear rate = velocity × 8/initial resting vessel diameter

### 2.6. Blood Sampling

Blood samples were drawn after an 8 to 12 h fast, while participants practiced stable dietary intake, medication use, and refrained from any moderate or strenuous physical activity other than the exercise completed for this study. Each experimental condition required three blood samples surrounding a single exercise session. Samples were obtained prior to exercise and 1-HR after exercise. Blood samples totaling 2.7 tbsp (40 mL) were obtained by venipuncture using a venous catheter or needle inserted into the most prominent vein site in the antecubital space (the arm that was not being used for brachial artery measurements was used to obtain blood samples). A total of two samples were drawn via the venous catheter during the first day of the experimental protocol. The catheter was maintained patent by introducing 0.5 mL of 10 USP/mL sodium-heparin flush after each of the first two samples. The venous catheter was removed after completing the last blood draw of the day. Another blood sample, equivalent to the other experimental samples, was obtained 24-HR after the exercise session using a 20- or 22-gauge needle. All blood samples were collected into red-top (no additive) and purple-top (KEDTA additive) vacuum-pressured specimen tubes.

A small amount of blood from each sample was immediately drawn into heparinized capillary tubes for estimating hematocrit [46]. Blood samples were placed on ice immediately after collection, and red-top specimens were allowed to clot. Samples were centrifuged at 3500 RPM for 15 min. Serum and plasma were recovered and aliquoted into storage tubes and stored at −80 °C until analysis.

### 2.7. Biochemical Analysis

Blood variables of interest related to nitrative stress influence on endothelial function are asymmetric dimethylarginine (ADMA) and 3-nitrotyrosine (^3−^NT). Paraoxonase-1 (PON1) and total antioxidant capacity (TAC) were measured to determine changes in antioxidant capabilities before and after exercise. Humoral epinephrine and norepinephrine were used to characterize physiological stress incurred during the exercise sessions. Enzyme-linked immunosorbent assays were performed to estimate changes in serum ADMA (MyBioSource, Inc., MBS264847, San Diego, CA, USA), ^3−^NT (abcam^®^, plc., ab116691, San Francisco, CA, USA), epinephrine and norepinephrine (R&D Systems, Minneapolis, MN, USA), PON1 activity (Zeptometrix, Catalog No. 0801199, Buffalo, NY, USA), and TAC (Cell Biolabs, Catalog No. STA-360, San Diego, CA, USA).

### 2.8. Statistics

Group physiological characteristics determined from screening assessments were summarized as means ± standard deviation. Significant differences in exercise were determined by two factor repeated measures ANOVA. The first factor, exercise condition, had two levels, high-intensity interval exercise and steady-state exercise. The second factor, sampling time, had three levels (pre-exercise, 1-HR, and 24-HR post-exercise) for FMD measurements and blood. Unadjusted measurements of flow-mediated dilation and those adjusted for shear rate indexed to pre-exercise resting arterial diameter were analyzed. Simple main effects were used to follow-up significant interactions.

We estimated the influence of baseline physiological variables, such as cardiovascular fitness, CKD status, by treating these measurements as covariates in the analysis of dependent variable changes. Within each condition, response effect sizes were calculated as mean differences from pre-exercise and using the pooled standard deviation between measurement points. Differences between changes in HIIE and SSE were calculated. Significance for all tests was set a priori at the *p* ≤ 0.05 level. Sample size was based on the FMD reproducibly paper by Welsch et al. [47]. All statistical procedures were carried out using SAS software version 9.4.

## 3. Results

### 3.1. Baseline Physiological Characteristics

Participant baseline physiological characteristics and blood variables are presented in Table 1. A total of 20 individuals, 14 females and 6 males, completed the study in a randomized crossover design. Participants were diagnosed with CKD stage IIIa or IIIb.

### 3.2. Flow-Mediated Dilation Outcomes

**Figure 2:**
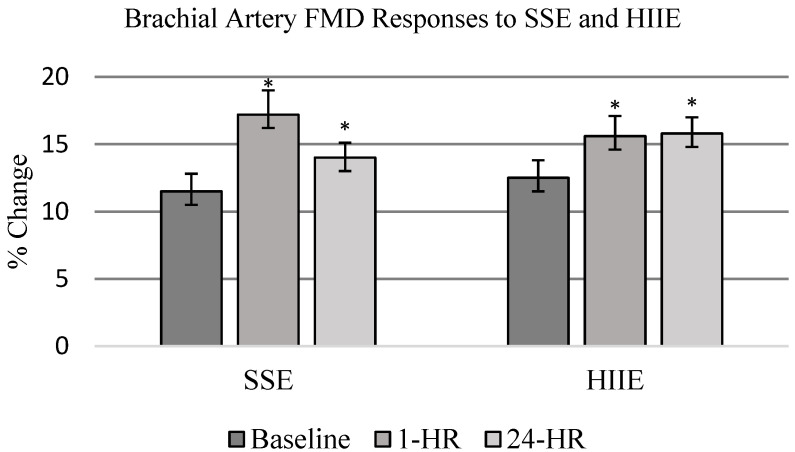
FMD responses to SSE and HIIE. FMD measurements were performed at baseline, 1 h post-exercise (PE), and 24 h PE. Data are presented as mean ± SE and represent percent change in vessel diameter. Exercise significantly increased vessel diameter as measured by FMD when compared to baseline. SSE (pre-exercise = 11.5 ± 1.3; 1 h = 17.2 * ± 1.8; 24 h = 14.0 * ± 1.1%) HIIE (pre-exercise = 12.5 ± 1.3; 1 h = 15.6 * ± 1.5; 24 h = 15.8 * ± 1.2%) with no statistically significant difference between exercise conditions (* *p* < 0.05 compared to baseline measure).

All participants completed and tolerated each FMD measurement point. There was no difference in resting vessel diameter pre-occlusion between HIIE and SSE (F_1,19_ = 0.19, *p* = 0.6647). FMD measurements performed after exercise increased in both HIIE and SSE conditions when compared to baseline measurements (F_2,18_ = 8.50, *p* = 0.0009) (Table 2). There were no significant differences between exercise conditions (F_2,18_ = 0.11, *p* = 0.747). When collapsed across conditions, FMD was significantly increased 1-HR and 24-HR post-exercise (F_2,18_ = 6.10, *p* = 0.005) (see Figure 2). Comparing 1-HR post-exercise to baseline resulted in a moderate effect size of 0.76. Twenty-four-hour post-exercise also had a moderate effect size of 0.55 when compared to baseline FMD measurements. Shear rate was calculated for each condition and reported in Table 3. In absolute terms, the shear rate remained unchanged when comparing conditions and time. When FMD was normalized to shear rate, there was a significant change (F_2,18_ = 8.46_,_ *p* = 0.0009) at 1-HR post-exercise with no difference observed between conditions. 

### 3.3. Biochemical Analysis Outcomes

**Figure 3:**
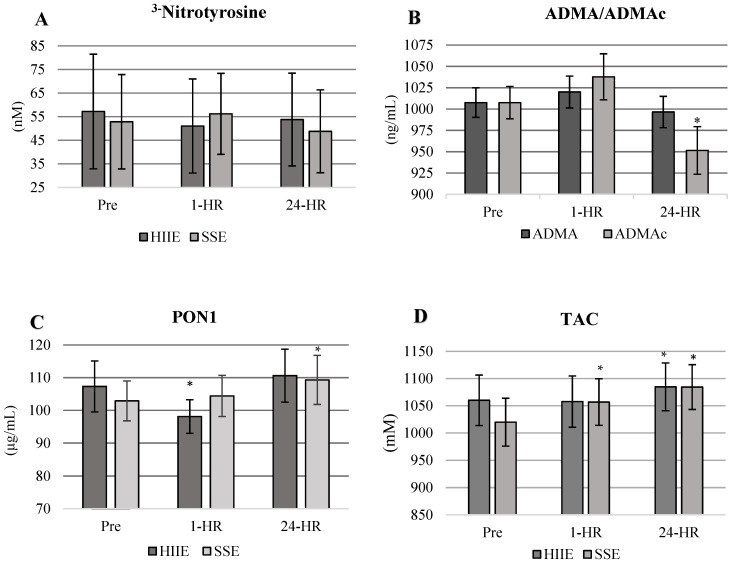
(**A**–**D**) Values are presented as means ± standard error. * = significantly different from Pre- to 1-HR and 24-HR post-exercise. (**A**) No significant differences in ^3−^NT were observed between conditions or time points. (**B**) ADMAc is significantly decreased in both conditions at 24-HR. Means are combined across conditions. ADMAc = Asymmetric dimethylarginine corrected for shifts in plasma volume. (**C**) PON1 response by condition and time. PON1 significantly increases at 24 h in SSE with no significant increases in HIIE. (**D**) TAC response by condition.

Biochemical analysis responses were analyzed as unadjusted and adjusted for plasma volume changes. Differences in ADMA, TAC, and PON1 were found between time points but no difference between conditions. No other differences were observed between the remaining variables. At 24-HR, ADMA concentrations were significantly reduced below baseline values in both conditions when correcting for shifts in plasma volume (*p* = 0.0006) (Figure 3B). There were no significant changes to ^3−^NT between conditions or across time (F_2,18_ = 1.64, *p* = 0.207). PON1 was increased across time (F_2,18_ = 5.24, *p* = 0.0097) for a condition by time main effect (F_2,18_ = 3.67, *p* = 0.0353) at 1-HR in HIIE and at 24-HR in SSE (Figure 3C). However, when corrected for shifts in plasma volume, PON1 had no significant changes between conditions across time (F_2,18_ = 2.91, *p* = 0.067). TAC increased in SSE at 1-HR and 24-HR and in HIIE at 24-HR post-exercise (F_2,18_ = 4.94, *p* = 0.012) (Figure 3D). TAC was not corrected to changes in plasma volume due to time-sensitive reagents required to analyze TAC.

## 4. Discussion

This study is the first to quantify the influence that an acute bout of aerobic exercise has on vascular endothelial function in the conduit arteries of individuals with mid-spectrum CKD. The key findings of the study are the following: (1) aerobic exercise improved vascular endothelial responsiveness; (2) regardless of exercise condition, the vascular endothelium responds positively to exercise as a stimulus; (3) markers of oxidative stress were decreased or unchanged and all antioxidant markers positively increased following exercise, regardless of exercise condition. The results support the utilization of aerobic exercise as a transient protective mechanism of vascular endothelial function in mid-spectrum CKD.

We hypothesized that a short bout of acute aerobic exercise would increase vascular endothelial function and that HIIE would have a greater influence on vascular endothelial function than SSE. Our hypothesis was based on improvements previously reported in vascular endothelial function in cardiometabolic cohorts similar to our participants [19,21,48]. In our study, vascular endothelial function was increased in the hours post-exercise regardless of exercise condition. FMD increased in HIIE (15.6% ± 1.5) and SSE (17.2 ± 1.8) at 1-HR post-exercise when compared to baseline (12%). When FMD means were collapsed across time, in a comparable amount, the average was 16.4%. Similar improvements in FMD were also observed at 24-HR with HIIE (15.8 ± 1.2) and SSE (14.0 ± 1.1). Improvements in vascular endothelial function after acute aerobic exercise are consistent with previously reported results, with two exceptions. In previous studies, HIIE had greater improvements in vascular endothelial function when compared to moderate-intensity exercise [19,47]. Both exercise interventions consisted of aerobic exercise on a cycle ergometer with either HIIE or moderate-intensity continuous exercise. While the individuals in these studies were diagnosed with CAD and accelerated cardiometabolic disease, the individuals in our study mainly had controlled hypertension and type II diabetes as their primary disease and mid-spectrum CKD as a secondary disease and were otherwise healthy. Additionally, increases in vascular endothelial function are traditionally limited to a transient period after exercise. Our study observed increases in vascular endothelial function at 24-HR post-exercise. Thus, the vascular endothelium may have responded differently to HIIE in our cohort compared to cohorts in previously reported studies. Baseline FMD was higher when compared to previous studies [41,49,50], which observed an FMD change of 2.5 to 7% at baseline. However, as previously noted, our population was healthier overall when compared to previous studies. Therefore, our cohort did not exhibit characteristics of endothelial dysfunction at baseline. Additionally, HIIE did not elicit as great of response in FMD when compared to healthy populations [25,51,52], indicating some level of endothelial impairment.

Markers of nitrative and oxidative stress and antioxidants were significantly altered following both HIIE and SSE. ADMA was significantly (*p* = 0.0006) decreased in both conditions post-exercise, indicating that the inhibition of eNOS was diminished (Figure 3B). Our responses in ADMA are similar to previously reported studies focused on acute bouts of aerobic exercise in healthy and diseased populations [53,54]. Aerobic exercise of moderate to high intensity has the potential to counter the excessive formation of oxidative and nitrative stress associated with elevated ADMA concentrations [55]. In previous studies, ADMA concentrations were reported to be 29% lower after completing 10 to 15 min of moderate-intensity aerobic exercise and 14% lower after a max exercise test [25,54,55]. Alterations in ADMA for our study appear to be time sensitive, with the most significant decreases being present at 24-HR. 

^3−^NT concentrations remained significantly unaltered in both HIIE and SSE conditions. Though, this response was not what we hypothesized. The lack of responsiveness in ^3−^NT appears to indicate that nitrative and oxidative stress was blunted in both exercise conditions. When ^3−^NT responses are coupled with the decrease in ADMA, the results support an increase in NO production, which was inferred by significant FMD responses in both HIIE and SSE. Potentially the vascular endothelium was ameliorated in the hours post-exercise.

Antioxidants PON1 and TAC significantly increased post-exercise (PON1 in SSE only and TAC in both conditions). PON1 was significantly altered in SSE; though it increased in HIIE, it was not enough to reach significance. TAC was increased in both HIIE and SSE; it appears SSE had the most significant influence in altering TAC. The greater increase in antioxidant capacity observed with PON1 and TAC with SSE may be the contributing factor to higher endothelial responsiveness in SSE at 1-HR when compared to HIIE. TAC at 24-HR post-exercise was comparable in both HIIE and SSE; however, at 1-HR post-exercise, there was no significant change to TAC in the HIIE condition. Antioxidant responses in our study are similar to studies reporting changes in PON1 and TAC after an acute bout of aerobic exercise of varying intensities and modalities in healthy and at-risk populations [56,57,58].

Limitations to the study include changes in vascular function, which were not assessed outside of 24-HR. It is possible that different results would have been observed if FMD had been evaluated at periods greater than 24-HR. However, given peak changes in FMD usually are not observed after 24-HR post-exercise, we believe our study outcomes were not affected. Medication timing/usage could have potentially influenced our outcomes depending on the type of medications that participants were prescribed by their medical provider. Potentially, sample size may have been a limitation of the project. However, though our sample size was not large, our numbers reflected the recommended sample size in clinical populations when utilizing FMD [43,47].

Practical application for the research outcomes addresses the benefit of utilizing HIIE or SSE to stimulate the vascular endothelium in moderate stages of CKD. The potential benefit is to prevent the endothelium from progressing in the severity of endothelial dysfunction by actively promoting the NO pathway via increased shear force on the endothelium. Potentially this mechanistic pathway could influence the endothelium of the kidneys to improve or maintain renal decline.

## 5. Conclusions

Our study is the first to quantify the transient improvements in vascular endothelial function in mid-spectrum CKD that occur similarly to healthy individuals free of cardiometabolic diseases. The present study identifies that an acute bout of aerobic exercise varying in mode and intensity, with an equal amount of work, can provide significant transient improvements in vascular endothelial function in mid-spectrum CKD. The positive improvements in antioxidant markers and a reduction in nitrative and oxidative stress support the benefits of implementing aerobic exercise in mid-spectrum CKD. Our results suggest that aerobic exercise can be utilized as an additional therapeutic intervention to attenuate, or potentially reverse endothelial dysfunction associated with mid-spectrum CKD. Our findings indicate that HIIE appears to be safe and achievable for individuals with mid-spectrum CKD. Aerobic exercise training of varying modes and intensities in mid-spectrum CKD may potentially augment the vascular endothelium and its relation to the prevention and maintenance of vascular renal health and function in mid-spectrum CKD.

## Figures and Tables

**Table 1:** Baseline physiological characteristics.

Variables	(*n* = 20)
Age (y)	62.0 ± 10
Height (cm)	167.1 ± 8.6
Weight (kg)	80.9 ± 15.8
BMI (kg/m^2^)	28.8 ± 4.3
Waist (cm)	98.9 ± 12.7
BF (%)	28.8 ± 12.7
Rest SBP (mmHg)	125.4 ± 10.7
Rest DBP (mmHg)	81.4 ± 5.1
Rest HR (bpm)	71.3 ± 11.5
Creatinine (mg/dL)	1.11 ± 0.2
eGFR	51.5 ± 6.5
Glucose (mg/dL)	117 ± 70.2
Total Cholesterol (mg/dL)	173.7 ± 36.3
LDL (mg/dL)	91.9 ± 28.8
HDL (mg/dL)	50.7 ± 15.2
Triglycerides (mg/dL)	155.9 ± 53.2
VO_2 max_ (mL/kg/min)	19.4 ± 4.6
**Medications**	**Users (total *n* = 20)**
ARB	3 (20)
ACE Inhibitor	6 (20)
α-Blocker	2 (20)
β-Blocker	4 (20)
Metformin	7 (20)
Statin	10 (20)
Steroids	2 (20)
T3/T4	3 (20)

Note: All values are presented as mean ± standard deviation along with minimum and maximum values, and ranges. BF % = Body fat percentage; BMI = Body mass index; DBP = Diastolic blood pressure; eGFR = Estimated glomerular filtration rate; HDL-C = High-density lipoprotein cholesterol; HR = Heart rate; LDL-C = Low-density lipoprotein cholesterol; SBP = Systolic blood pressure; TC = Total cholesterol; TG = Triglycerides; Waist = Waist circumference.

**Table 2:** FMD results.

Pre			1-HR		24-HR	
Condition	Pre-OCC	Post-OCC	Pre-OCC	Post-OCC	Pre-OCC	Post-OCC
HIIE (mm)	3.42 ± 0.13	3.83 ± 0.12	3.44 ± 0.13	3.96 ± 0.13 *	3.35 ± 0.12	3.86 ± 0.13 *
SSE (mm)	3.37 ± 0.13	3.75 ± 0.12	3.33 ± 0.13	3.87 ± 0.11 *	3.43 ± 0.13	3.89 ± 0.13 *

Note: Values are presented as mean ± standard error. No significant differences were found between exercise conditions. Significant differences were observed across time for both conditions. Pre = before exercise; 1-HR = 1 h after exercise; 24-HR = 24 h after exercise. Pre-OCC = before cuff occlusion; Post-OCC = after cuff occlusion. The minimum pre-diameter was subtracted from the maximum post-diameter divided by minimum pre- diameter to calculate the % change in vessel diameter.

**Table 3:** Shear rate responses.

Condition	Pre	1-HR	24-HR
HIIE			
Max Flow (cm^−1^)	171.9 ± 11.7	173.5 ± 16.8	195.9 ± 14.2
Shear Rate (s^−1^)	374.7 ± 35.2	370.9 ± 52.2	429.1 ± 41.6
SSE			
Max Flow (cm^−1^)	172.9 ± 10.5	154.3 ± 9.3	165.9 ± 9.3
Shear Rate (s^−1^)	373.8 ± 22.4	325.5 ± 23.5	351.4 ± 26.6

Note: Values are presented as mean ± standard error. Shear rate = 8 × max diameter/min diameter. No significant differences were found between exercise conditions or across time.
